# Rhabdomyosarcome paratesticulaire: à propos d’un cas et revue de la littérature

**DOI:** 10.11604/pamj.2021.39.71.29224

**Published:** 2021-05-26

**Authors:** Boris Amougou, Divine Eyongeta, Jean Paul Engbang, Theodore Sala Beyeme, Demba Cisse, Marcel Jerry Ngandeu, Yaya Sow, Abdoulaye Bobo Diallo

**Affiliations:** 1 Département de Chirurgie et Spécialités Chirurgicales, Faculté de Médecine et des Sciences Pharmaceutiques, Université de Dschang, Dschang, Cameroun,; 2 Départements d´Anatomie et de Chirurgie, Faculté de Médecine, Université de Buea, Département d’Urologie, Hôpital Régional de Limbé, Limbé, Cameroun,; 3 Faculté de Médecine et des Sciences Pharmaceutiques de l´Université de Douala, Douala, Cameroun,; 4 Service d´Urologie Hôpital Laquintinie de Douala, Douala, Cameroun,; 5 Faculté de Médecine et de Pharmacie de l´Université Gamal Abdel Nasser de Conakry, Conakry, Guinée,; 6 Faculté de Médecine, Pharmacie et Odonto-Stomatologie, Université Cheikh Anta Diop, Service d´Urologie, Hôpital Aristide Le Dantec, Dakar, Sénégal,; 7 Département de Chirurgie et Spécialités Chirurgicales, Faculté de Médecine et de Pharmacie de l´Université Gamal Abdel Nasser de Conakry, Conakry, Guinée

**Keywords:** Rhabdomyosarcome, testicule, à propos d’un cas, Rhabdomyosarcoma, testicle, case report

## Abstract

Nous rapportons une observation d´un rhabdomyosarcome embryonnaire paratesticulaire chez un adulte jeune et soulignons le caractère inhabituel de cette forme histologique dans cette tranche d´âge, l´évolution rapide de la lésion ainsi que les difficultés de prise en charge de ce type de tumeurs dans notre contexte.

## Abstract (en)

We here report a case of embryonal paratesticular rhabdomyosarcoma in a young adult. The purpose of this study is to highlight this uncommon histological type of tumor in this age group, the rapid evolution of the lesion and the challenges of managing it in our context.

## Introduction

Le rhabdomyosarcome (RMS) est une tumeur mésenchymateuse maligne rare développée aux dépens des tissus conjonctifs dont les localisations génito-urinaires sont les plus fréquentes [1]. La localisation paratesticulaire représente 7% de l´ensemble des rhabdomyosarcomes, toutes localisations confondues [2, 3]. L´âge de survenue est caractérisé par deux pics d´incidence, le premier entre 2 et 5 ans, le second à l´adolescence [4]. Cela explique la fréquence élevée des deux formes histologiques majeures des rhabdomyosarcomes : embryonnaire et alvéolaire. L´incidence de la forme embryonnaire est plus élevée à la naissance et se prolonge pendant la petite enfance pour diminuer à l´adolescence [5, 6]. La présentation clinique n´est pas spécifique et le diagnostic est le plus souvent fait à l´examen anatomopathologique de la pièce opératoire d´orchidectomie [7]. La prise en charge multidisciplinaire, est fonction du stade clinique et du groupe pronostic de l´International Rhabdomyosrcoma Society (IRS) et associe chirurgie, chimiothérapie et parfois radiothérapie. Nous rapportons une observation de rhabdomyosarcome paratesticulaire (RPT) de type embryonnaire caractérisée par sa survenue inhabituelle chez un adulte jeune et en discutons les aspects cliniques et thérapeutiques dans notre contexte à la lumière d´une revue de la littérature.

## Patient et observation

**Motifs de consultation et antécédents médicaux:** BB, 22 ans, sans antécédents médicaux ni chirurgicaux personnels ou familiaux particuliers a consulté notre service pour une grosse bourse droite d´apparition spontanée, qui a progressivement augmentée de volume depuis 6 mois, accompagnée de douleurs sourdes sans signes urinaire ni général associés.

**Figure 1:**
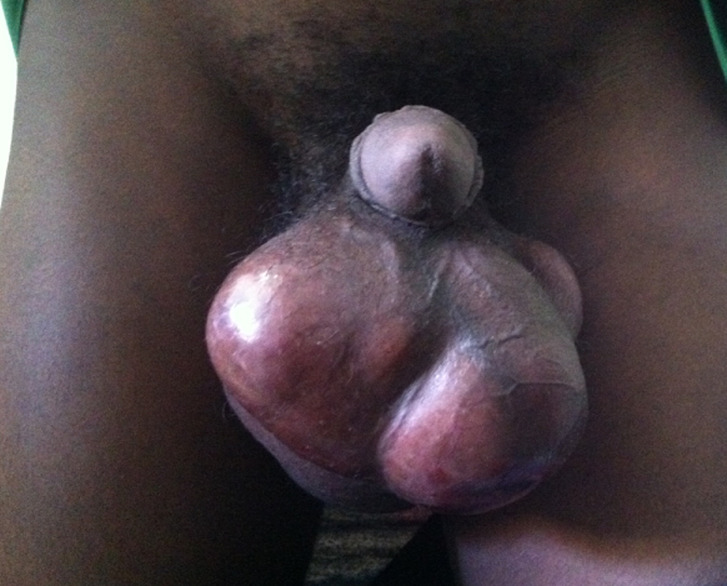
aspect de la bourse à l´examen

**Examen physique:** l´examen clinique a révélé chez un patient en bon état général et avec des constantes vitales normales, une volumineuse tuméfaction scrotale droite, de consistance élastique, polylobée, irrégulière, sensible avec une peau scrotale tendue et luisante. Un épaississement du cordon spermatique ipsilatéral sans adénopathie inguinale palpée (Figure 1). Le testicule controlatéral était palpé sans anomalies. L´examen des autres appareils étaient normal.

**Bilan complémentaire:** l´échographie scrotale effectuée a révélé une masse tissulaire de 10 cm développée soit aux dépens de l´épididyme soit du testicule soit des enveloppes testiculaires refoulant le testicule gauche qui est par ailleurs normal. Le dosage des marqueurs tumoraux alpha-foetoprotéine, lactate déshydrogénase et l´hormone chorionique gonadotrope humaine totale était normal. Au terme de ce bilan nous avons évoqué le diagnostic d´une tumeur du testicule droite.

**Figure 2:**
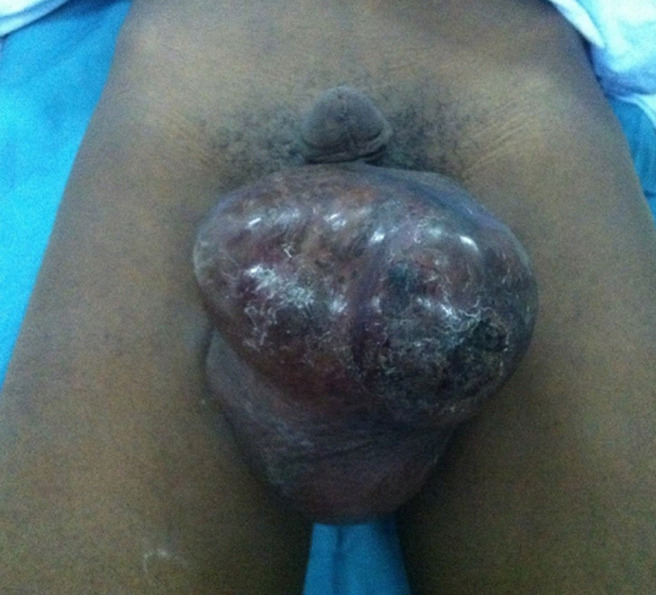
aspect préopératoire (deux semaines après la consultation initiale)

**Figure 3:**
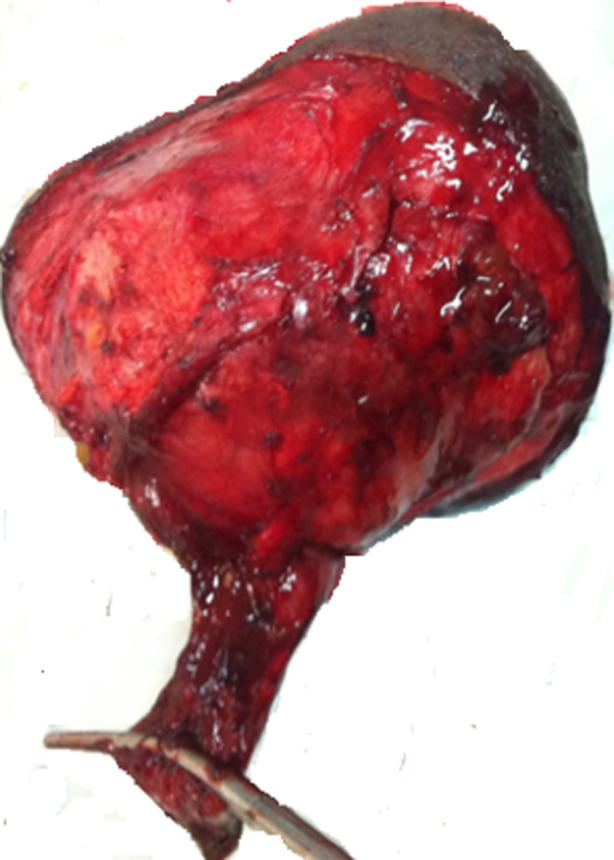
aspect de la pièce opératoire d´hémiscrotectomie

**Prise en charge:** une orchidectomie droite première a été décidée en réunion de concertation pluridisciplinaire mais devant l´aspect de la lésion en préopératoire (Figure 2), une hémiscrotectomie droite avec ligature première haute du cordon a été réalisée sans curage ganglionnaire inguinale (Figure 3).

**Suivi postopératoire:** les suites opératoires immédiates et à distance ont été simples. L´examen anatomopathologique de la pièce d´exérèse a révélé un rhabdomyosarcome embryonnaire paratesticulaire de 13 cm de grand axe avec une peau scrotale ulcérée. Le patient a été revu à un mois postopératoire avec une tomodensitométrie thoraco-abdomino-pelvienne (TDM TAP) comme bilan d´extension qui a révélé une lésion nodulaire pulmonaire unique, isolée dans le segment postéro-basal du poumon gauche pouvant correspondre à une localisation néoplasique secondaire sans autres anomalies suspectes dans les étages abdominal et pelvien. L´examen clinique révélait un patient en bon état général sans anomalie décelée. Une surveillance (clinique et radiologique par tomodensitométrie) avait été décidée en RCP compte tenu des moyens financiers limités. Le patient est perdu de vue après le premier contrôle post opératoire.

## Discussion

Le rhabdomyosarcome paratesticulaire est une entité rare représentant environ 7% de tous les rhabdomyosarcomes pédiatrique [8]. Il s´agit d´une tumeur rare caractérisée par une évolution rapide et un pronostic mauvais. A notre connaissance il s´agit du premier cas décrit en milieu hospitalier au Sénégal. Dans la littérature, la majorité des publications sur le rhabdomyosarcome paratesticulaire traitent des cas isolés [9, 10]. L´âge de survenue est caractérisé par deux pics d´incidence, le premier entre 2 et 5 ans, le second à l´adolescence [4]. Notre cas présente la particularité qu´il s´agissait d´une forme histologique du petit enfant survenue chez un adulte jeune. Sur le plan clinique, la grosse bourse douloureuse a été le mode de présentation de la maladie ayant conduit le patient a consulté. Il s´agit en effet du mode de présentation habituelle retrouvé dans la littérature [11]. L´examen physique de la bourse de notre patient nous a permis d´éliminer une torsion testiculaire et une orchiépididymite, par contre le testicule droit n´était pas parfaitement identifiable lors de cet examen. De même le siège paratesticulaire de la masse était difficile à préciser. Nos constations sont conformes aux données retrouvées dans la littérature. Certains auteurs notent que devant une bourse aigue le diagnostic est parfois difficile du fait de la présentation souvent confondue avec une torsion testiculaire et que le siège paratesticulaire de la masse est difficile à préciser par la palpation du scrotum, le testicule étant difficilement identifiable [8, 12].

Sur le plan paraclinique, la suspicion d´une tumeur testiculaire impose la réalisation systématique d´une échographie scrotale en raison de sa sensibilité élevée proche de 100% [13]. Cet examen objective une masse de densité tissulaire, hétérogène, intrascrotale, développée aux dépens des enveloppes testiculaires, le testicule est le plus souvent normal. Dans notre cas, peut-être en raison du volume important de la masse scrotale, l´échographie scrotale n´a pas été contributive ne permettant pas de localiser le siège de la tumeur. Néanmoins si l´échographie confirme la présence d´une tumeur les marqueurs tumoraux doivent être réalisés avant toute prise en charge. Ceux-ci sont en général normaux dans les tumeurs embryonnaires et il n´existe en réalité pas de marqueurs tumoraux pouvant aider au diagnostic qui repose essentiellement sur l´examen anatomopathologique de la pièce d´orchidectomie réalisé par voie inguinale mettant en évidence des rhabdomyoblastes caractéristique du rhabdomyosarcome [11]. La tomodensitométrie thoraco-abdomino-pelvienne réalisée dans le cadre du bilan d´extension de notre patient avait révélé une lésion nodulaire pulmonaire unique, isolé dans le segment postéro-basal du poumon gauche. L´extension à distance se fait par voie lymphatique et hématogène. Les ganglions rétropéritonéaux représentent le premiers relais ganglionnaire [14]. Le poumon, le foie et l´os sont les sites métastatiques les plus fréquents. Cet examen bien qu´indispensable dans la stratégie thérapeutique ne doit cependant pas retarder la prise en charge, de ce fait est parfois réalisé après l´orchidectomie comme ce fut le cas chez notre patient. D´autres examens d´imagerie peuvent également en cas de doute diagnostic être prescrits c´est le cas de l´imagerie par résonnance magnétique (IRM) et de la tomographie par émission de positons (PET-scan). Ils ne sont cependant pas accessibles à tous nos patients en raison de leur cout élevé. Sur le plan histologique, trois formes de rhabdomyosarcome existent: la forme embryonnaire (la plus fréquente, 97% des cas), la forme alvéolaire et la forme pléomorphe. L´extension locale est très précoce (comme ce fut le cas chez notre patient avec l´envahissement scrotal survenu 2 semaines après l´examen initial). Le rhabdomyosarcome embryonnaire est de mauvais pronostic du fait de l´atteinte ganglionnaire observée dans 40% des cas au moment du diagnostic [15], néanmoins la majorité des auteurs s´accordent sur le fait que la précocité du diagnostic et du traitement chirurgical conditionne le pronostic [8].

Sur le plan thérapeutique, la stratégie thérapeutique dépend du stade de la tumeur et du groupe pronostique selon la classification de l´International Rhabdomyosarcoma Society [12]. La prise en charge doit être multidisciplinaire et décidée en réunion de concertation pluridisciplinaire(RCP). Les options thérapeutiques en plus de l´orchidectomie sont le curage lombo-aortique, la chimiothérapie et la radiothérapie. Le stade tumoral de notre patient après le bilan d´extension était T1N0M1 et le patient a été classé dans le Groupe I de l´International Rhabdomyosarcoma Society (IRS) [12] (Tableau 1), de ce fait il a eu une orchidectomie, réalisée par voie inguinale avec ligature première haute du cordon spermatique tel que recommandé. Nous n´avons pas réalisé de curage ganglionnaire lombo-aortique en raison de l´absence d´atteinte ganglionnaire radiologique. Depuis la publication de la Société Internationale d´Oncologie Pédiatrique (SIOP), ce curage ganglionnaire para-aortique systématique est controversé [15] et son intérêt dans les formes localisées est discuté en raison de l´efficacité de la chimiothérapie sur les micrométastases ganglionnaires [16]. En outre la morbidité non négligeable de ce curage doit être mise en balance avec le risque de récidive, de ce fait il est actuellement recommandé de ne pas réaliser de lymphadénectomie en l´absence d´atteinte ganglionnaire mise en évidence par la tomodensitométrie. La chimiothérapie, bien qu´indiquée dans tous les stades n´a pas été réalisée chez notre patient en raison du cout élevé des drogues utilisées qui sont laissées à la poche de patients souvent démunis. Différents protocoles sont disponibles avec des durées variant de 18 à 24 mois. Ce sont le protocole VAC, IVA et VIE (V) vincristine; A) actinomycine D; E) étoposide; I) ifosfamide et C) cyclophosphamide). Le protocole VAC est le plus utilisé. Dans des indications limitées, la radiothérapie est réalisée en complément de la chimiothérapie et du curage sur les foyers tumoraux résiduels, sur les ganglions rétropéritonéaux et sur les métastases en particulier pulmonaires. Elle n´a pas été réalisée chez notre patient en raison du doute sur la nature de la lésion mise en évidence à la tomodensitométrie thoracique ajoutée à cela l´insuffisance de notre plateau technique qui ne permet pas de délivrer des doses contrôlées d´irradiation à un volume cible.

**Tableau 1:** classification des rhabdomyosarcomes selon l’Intergroup Rhabdomyosarcoma Study (IRS)

Groupe	Caractéristiques
**Groupe I: tumeur localisée, réséquée complètement, marges négatives et pas d’atteinte ganglionnaire régionale**	a) confinée au muscle ou à l’organe d’origine
	b) extension locale avec infiltration hors du muscle ou de l’organe d’origine
**Groupe II: exérèse macroscopique complète, mais extension régionale**	a) exérèse macroscopique complète mais pas de résidu microscopique
	b) atteinte ganglionnaire complètement réséquée (atteinte des lymphonoeuds régionaux et/ou extension de la tumeur vers un organe adjacent ; pas de résidu microscopique)
	c) atteinte ganglionnaire : exérèse macroscopiquement complète mais résidu microscopique ou atteinte microscopiquement prouvée des ganglions les plus distaux du curage
**Groupe III**	**exérèse incomplète ou biopsie avec résidu microscopique**
**Groupe IV**	**métastases à distance au diagnostic**

NB : la classification se répartit en 4 groupes en fonction des résultats chirurgicaux et de l’extension tumorale. Cette classification s’intéresse à tous les rhabdomyosarcomes toutes localisations et types histologiques confondus.

Le pronostic du rhabdomyosarcome paratesticulaire dépend du stade tumoral, du type histologique et de la réponse au traitement. Toutefois, la survie globale des rhabdomyosarcomes de type embryonnaire est de 78%, toutes variables confondues [17]. Le risque d´évolution tumorale est également lié à l´âge. Les formes de l´adolescent et de l´adulte sont de plus mauvais pronostic. Le suivi post-thérapeutiques des patients traités pour rhabdomyosarcome paratesticulaire est clinique, biologique et radiologique et doit être poursuivi à vie.

***Leçons à retenir:*** ce cas clinique présente la particularité épidémiologique d´être le premier cas de rhabdomyosarcome paratesticulaire décrit en milieu hospitalier au Sénégal. Sur un plan diagnostic, l´échographie scrotale n´a pas été déterminante car elle n´a pas permis de discriminer cette masse scrotale et les marqueurs tumoraux biologiques n´ont pas permis de trancher. Comme devant toute suspicion de tumeur testiculaire et l´absence d´imagerie tomodensitométrique dans l´immédiat, l´orchidectomie première après ligature du cordon spermatique est la règle et constitue le premier temps de la prise en charge. Elle s´est d´autant plus justifiée dans notre cas clinique que l´évolution en seulement deux semaines a été spectaculaire. L´insuffisance des moyens financiers a considérablement limité les options thérapeutiques et le suivi optimal de notre cas.

**Consentement éclairé du patient:** le patient a reçu une information éclairé de son diagnostic et des options thérapeutiques adaptées au stade de sa maladie et a consenti à l´intervention chirurgicale proposée.

## Conclusion

Le rhabdomyosarcome paratesticulaire est une tumeur rare, la forme embryonnaire est de mauvais pronostic lorsqu´elle survient à un âge avancé. Un diagnostic précis et un traitement précoce sont les garants d´une meilleure survie. La stratégie thérapeutique est bien codifiée et dépend du stade tumoral et du groupe pronostique. Le pronostic des formes vues tardivement est rapidement mauvais. A la lumière de ce premier cas décrit en milieu hospitalier au Sénégal et des limites notées dans la prise en charge de ce patient nous pensons qu´une meilleure politique de santé en matière de cancer permettrait, par le billet de subventions des drogues, de les rendre accessible à tous nos patients et permettrait certainement d´améliorer la survie globale de ces patients.
